# Cryopreservation of hepatocytes: Impact of long-term storage and organ donor factors on cell quality for clinical applications

**DOI:** 10.1177/09636897261417050

**Published:** 2026-04-19

**Authors:** Kadriye Güven, Valeria Iansante, Sharon C. Lehec, Ragai R. Mitry, Anil Dhawan, Céline Filippi

**Affiliations:** 1 DhawanLab, Institute of Liver Studies, King’s College London, King’s College Hospital, London, UK; 2 Paediatric Liver, GI and Nutrition Centre, King’s College London, King’s College Hospital, London, UK

**Keywords:** primary human hepatocytes (PHHs), hepatocyte transplantation, cryopreservation, donor age, cold ischemia time (CIT), body mass index (BMI)

## Abstract

Liver transplantation is the only curative option for end-stage liver disease, but its applicability is limited by donor shortages. Hepatocyte transplantation offers a promising alternative, particularly for pediatric acute liver failure, yet its success relies on the availability of viable cells. Cryopreservation enables off-the-shelf use, though long-term storage may impair cell quality. This study investigates whether cryostorage duration, donor characteristics, and organ retrieval parameters affect the viability and functionality of cryopreserved primary human hepatocytes (PHHs). We conducted a retrospective analysis of 144 thawing events across 81 GMP-grade hepatocyte batches, cryopreserved for up to 14 years. Donor age ranged from 3 days to 70 years. Viability and functionality were assessed using MTT assays, albumin and urea secretion, and CYP450 activity. Linear regression was used to analyze correlations (*P* < 0.05 considered significant). The cryopreservation duration had no significant impact on hepatocyte viability or function, confirming that clinical-grade PHHs can maintain quality for up to 14 years. However, donor age correlated negatively with post-thaw viability (*R*^2^ = 0.035, *P* = 0.029), yield (*R*^2^ = 0.039, *P* = 0.041), and metabolic activity (*R*^2^ = 0.278, *P* = 0.008), while showing a positive correlation with induced ethoxy resorufin O-demethylation (EROD) function (*R*^2^ = 0.432, *P* = 0.020). Donor body mass index (BMI) showed significant negative associations with viability (*R*^2^ = 0.075, *P* = 0.003), yield (*R*^2^ = 0.069, *P* = 0.011), and metabolic activity (*R*^2^ = 0.267, *P* = 0.019). Cold ischemia time (CIT) showed a weak negative correlation with viability (*R*^2^ = 0.151, *P* < 0.0001), particularly when CIT exceeded ~10 h and viability fell below 60%. Clinical-grade cryopreserved hepatocytes retain viability and function for over a decade. CIT, donor BMI, and age impacted post-thaw quality, while warm ischemia time (WIT), within our current criteria, and storage duration had minimal impact. These findings support refining donor selection to improve cryopreservation outcomes.

## Graphical abstract

**Graphical Abstract:**
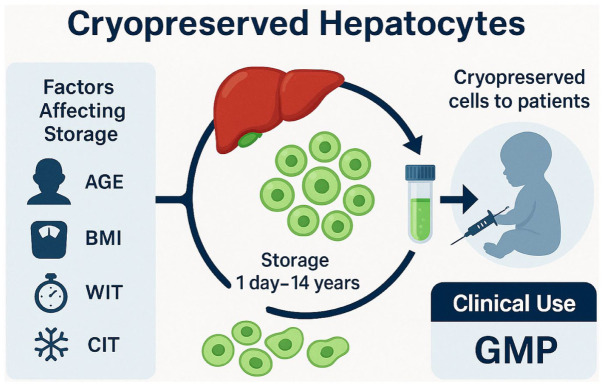
This is a visual representation of the abstract.

## Introduction

The liver is renowned for its remarkable regenerative capacity following injury. However, in late-stage liver diseases where regeneration is no longer possible, liver transplantation is the only curative option. In cases of pediatric acute liver failure, the liver’s regenerative capacity has been demonstrated through auxiliary liver transplantation, with 70% of recipients successfully regenerating their native liver. This showed that, despite being lifesaving, whole organ transplantation may not be the most ideal treatment option^1–5^. As a promising alternative, isolated primary human hepatocyte (PHH) transplantation has emerged as a potential alternative for treating liver diseases^6–8^. Hepatocyte isolation from a single donor liver can yield billions of cells, sufficient to treat multiple patients. This approach aims to restore liver function or repair damaged tissue by transplanting viable hepatocytes directly into the patient’s liver, where they would engraft and expand to reach a critical mass to ameliorate the patient’s condition. For acute liver failure cases, hepatocyte transplantation could also serve as a bridge therapy, supporting patients until their native liver recovers or a suitable donor organ becomes available^9–13^.

Hepatocyte transplantation holds significant potential but faces critical challenges, particularly in securing a sufficient supply of high-quality hepatocytes when they are needed. Freshly isolated PHHs are often unavailable for immediate use in acute liver injuries or end-stage liver disease, where off-the-shelf cell therapies would be ideal. Cryopreserved hepatocytes provide a practical solution, but their therapeutic efficacy is compromised by the detrimental effects of cryopreservation and thawing. Previous studies have shown that cryopreservation could negatively impact hepatocyte viability and function, leading to freeze-thaw damage, reduced cell viability, early apoptosis, and impaired functionality, such as diminished albumin production and engraftment potential^9,14,15^. In addition to cryopreservation, the donor’s health status plays a crucial role in hepatocyte isolation, directly influencing sample quality and viability. Research has shown that donor-associated factors such as body mass index (BMI) and donor age significantly affect hepatocyte yield and viability, as do organ retrieval details of warm ischemia time (WIT) and cold ischemia time (CIT)^1,16–20^. Cryopreserved primary hepatocytes have been used in several studies for the treatment of pediatric liver diseases, with some reports indicating comparable therapeutic potential to freshly isolated cells^2^. In these studies, PHHs had been cryopreserved for up to 5 years; however, it remains unclear whether extended storage duration affects cell quality. Notably, the cells used in some cases may have only been stored for 1–2 years, and further investigation is needed to determine the impact of long-term cryopreservation on cell viability and function.

This study explores the effects of cryostorage duration on hepatocyte quality markers. It also examines the effects of donor characteristics and organ retrieval parameters on post-thaw hepatocyte viability and functionality. By investigating these factors, we aimed to provide (1) the necessary information to determine hepatocyte shelf life for clinical use and (2) refine our organ specifications for long-term cell storage.

## Methods

### Study design

Donor liver tissue unsuitable for transplantation was obtained for hepatocyte isolation following the ethical approval consented by The Research Ethics Committee of King’s College Hospital, which was already in place (LREC 01-016). A retrospective analysis was carried out on the hepatocyte batches cryopreserved between 2002 and 2024 and thawed between 2016 and 2024 by the hepatocyte group at King’s College Hospital. The analysis included up to *n* = 144 cell thawing performed on *n* = 81 batches of hepatocytes, with a storage time between 1 day and 14 years. The age of donors was between 3 days and 70 years. The age of cells at thawing (storage time) refers to the duration between the initial freezing of the hepatocyte cells and their thawing for experimental analysis. This was calculated in days (specify the units) based on the documented freezing and thawing dates recorded in the cell storage logs. Donor-related information was recorded and obtained directly from donor medical records. WIT refers to the duration between the cessation of blood flow to the liver and the initiation of cold preservation^21^. WIT was measured in minutes and documented during the harvesting process by trained personnel. CIT represents the period between the start of cold preservation and the beginning of cell isolation^22^. CIT was recorded in hours and tracked using a standardized protocol. BMI was obtained from medical records. It should be noted that not all cryopreserved hepatocyte batches were analyzed for the same parameters over the same period. While viability and total cell yield data were available for batches cryopreserved for up to 14 years, the dataset for additional assays was limited to samples stored for 5–10 years.

### Cell culture and preparation

#### Cell isolation, cryopreservation, and thawing

**Figure 1.**
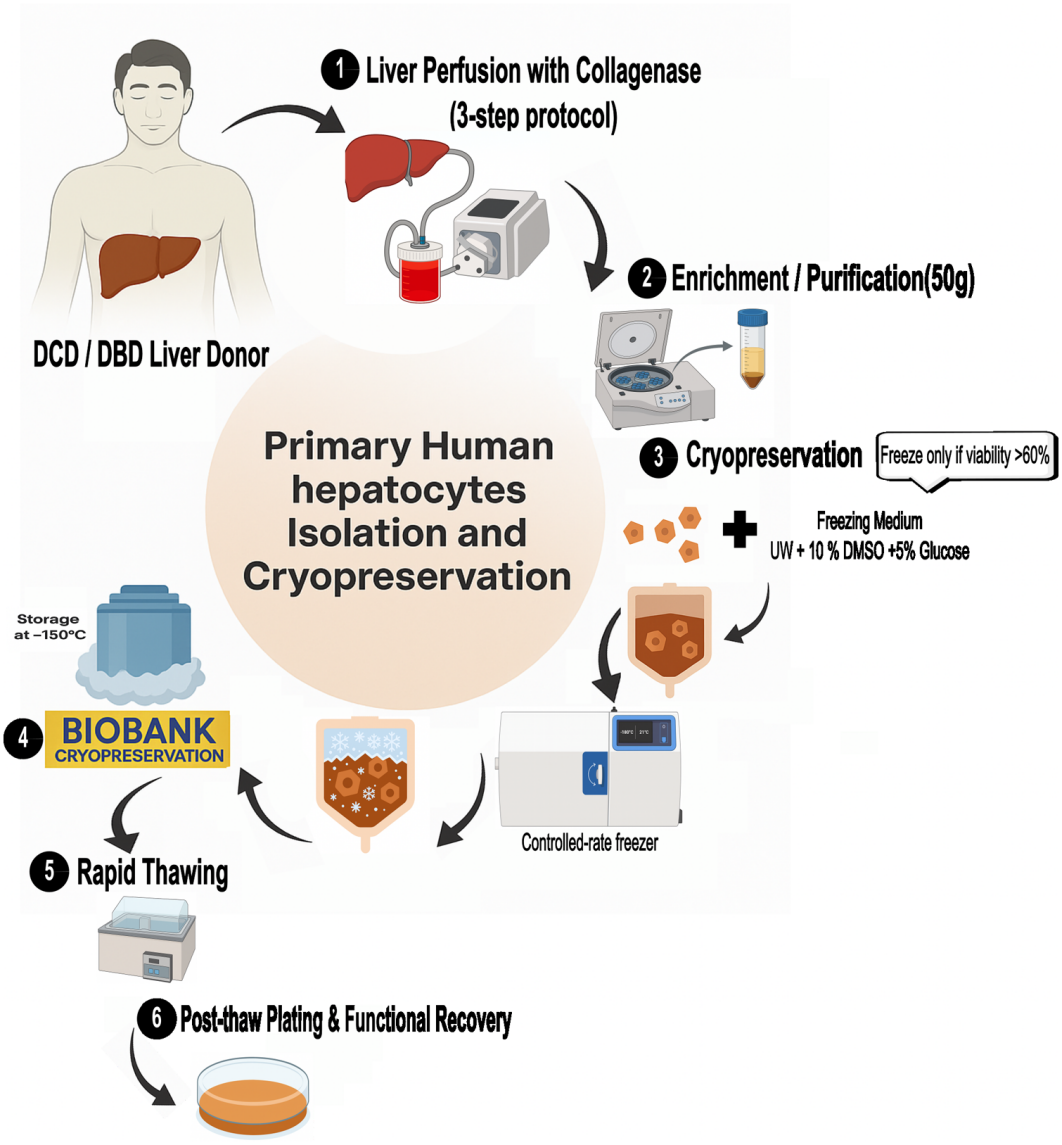
Schematic overview of primary human hepatocyte isolation, cryopreservation, and thawing workflow. Donor livers (DCD/DBD) were processed using collagenase perfusion, cell enrichment, and cryopreservation (≥60% viability) in UW solution with 10% DMSO and 5% glucose. Cells were stored at −150°C and, upon rapid thawing, assessed for post-thaw functional recovery (albumin, urea, CYP activity).

PHHs were isolated from donor liver tissue samples (rejected or unused for transplantation) using a modified version of the three-step collagenase perfusion method^23^. After a viability assessment, hepatocytes were resuspended at 10^7^ cells/ml in the following cryopreservation solution: University of Wisconsin (UW) solution (Bristol-Myers Squibb AB, Sweden), supplemented with 10% dimethyl sulfoxide (DMSO; WAK-Chemie Medical GmbH, Germany) and 5% glucose (Hameln Pharmaceuticals Ltd., Gloucester, UK), before being cryopreserved in a controlled-rate freezer, as described in previous work^24^. The hepatocytes were then stored at below −130°C. When needed, the cells were thawed using a rapid thawing technique^25^. A schematic overview of the hepatocyte isolation, cryopreservation, and thawing workflow is shown in Fig. 1.

#### Cell viability assay

Viability and cell yield were assessed before cryopreservation using Trypan Blue exclusion (Sigma-Aldrich, Irvine, UK). The initial viability of freshly isolated hepatocytes was recorded, along with the total cell yield per donor (*n* = 144). Cell batches were cryopreserved only when their viability was equal to or higher than 60%.

Viability after thawing was also determined directly after cell thawing. Cell recovery was calculated as the percentage of total cells retrieved after thawing and centrifugation relative to the initial number of cryopreserved cells.

#### MTT assay for oxidative function

Cell oxidative function was evaluated using the MTT assay (Sigma-Aldrich, Irvine, UK). Viability was assessed on day 1 following cell thawing. Briefly, cells with viability exceeding 60% were seeded into a 96-well plate and incubated at 37°C in a 5% CO₂ environment. After treatment, MTT solution (5 mg/ml in William’s E medium) was added to each well and incubated for 2–4 h at 37°C. The resulting formazan crystals were dissolved by adding DMSO. Absorbance was then measured at 570 nm using a microplate reader.

#### Hepatic-specific functional assays

Biosynthetic capacity was assessed by measuring the production of hepatocyte-specific proteins albumin and α1-antitrypsin (AAT). Human albumin and AAT secreted by the cells into the culture medium over 24 h were quantified using enzyme-linked immunosorbent assay (ELISA) Quantitation Kits (Bethyl Laboratories, TX, USA), following the manufacturer’s instructions.

Urea synthesis was measured after washing cultured cells twice with phosphate-buffered saline (PBS; Gibco, UK) and incubating them in a serum-free medium with 5 mM ammonium chloride for 4–6 h. The supernatant was then collected and analyzed using the QuantiChrom Urea Assay Kit (Universal Biologicals, Cambridge, UK), following the manufacturer’s instructions. In a small set of samples, hepatic drug metabolism was assessed by measuring the activity of CYP1A1/2. CYP1A1/2 activity and its inducibility were evaluated using the ethoxy resorufin O-demethylation (EROD) assay. In this assay, cells were treated for 72 h with omeprazole (50 μM) as an inducer. Their ability to metabolize 7-ethoxy resorufin (5 µM in William’s E medium) to fluorescent resorufin in the presence of salicylamide (1.5 mM) was assessed after 4 h of incubation at 37°C. A plate reader was used to detect the resorufin fluorescence at an excitation/emission of 544/584nm. A standard curve of resorufin was used to quantify the results. In separate wells, phase 2 activity was evaluated by measuring resorufin metabolism. Cells were incubated with 50 ng/ml of resorufin for 2 h at 37°C. As resorufin undergoes conjugation by phase 2 enzymes, it loses its fluorescence. Phase 2 metabolism was thus quantified as a decrease in resorufin fluorescence and expressed as nanograms of conjugated resorufin formed per hour.

All functional assays (CYP450, MTT, albumin, and urea) were performed under standardized conditions using the same equipment, reagents, and culture settings (37°C, 5% CO_₂_) and appropriate normalization. Procedural updates during the study period were applied uniformly across all batches.

### Statistical analysis

The dependent and independent variables were represented in a scatter plot using GraphPad Prism 7.01 software. A linear regression analysis was used (95% confidence interval). The correlation coefficient *r*^2^ and *P*-value were measured. In any condition where *P* < 0.05, the correlation coefficient was considered statistically significant. Sample sizes (*n*) varied because this was a retrospective study aggregating data obtained by various scientists in our lab; while they followed the same protocols, not all researchers performed every assay available in the laboratory. Consequently, the low *n* number for some functional activities resulted in reduced statistical power and more limited conclusions for those assays. Exact *n* values are provided in each figure legend.

## Results

### Donor demographics and ischemia times

**Figure 2.**
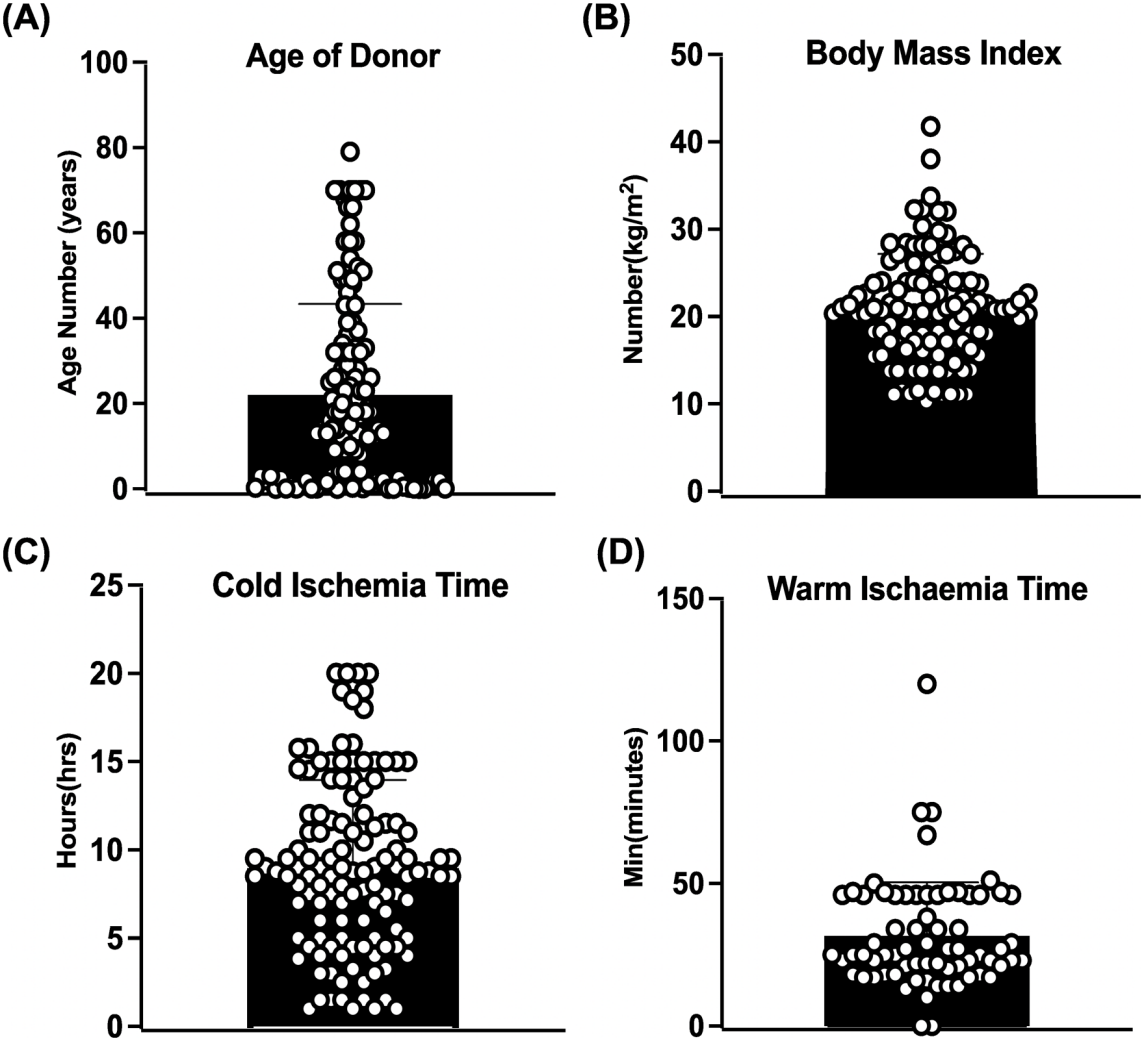
(a) Donor age, (b) body mass index (BMI), (c) cold ischemia time, and (d) warm ischemia time. Each open circle represents an individual donor, and the black bar indicates the mean ± standard deviation (SD). The distributions observed in all four measures underscore the widespread nature of the donor pool. Data were collected from *n* = 81 donors, with multiple samples analyzed across various cryopreservation times.

Figure 2 summarizes key donor/tissue characteristics. Fig. 2a illustrates the donor age distribution, which ranged from 3 days to 79 years, with a mean age of 22.0 ± 21.2 years. A significant proportion of donors (53%) were younger than 18 years of age, highlighting a strong pediatric contribution. Fig. 2b shows donor BMI values, which range from 10.29 to 41.78 kg/m^2^, with a mean of 20.75 ± 6.17 kg/m^2^. This wide distribution reflects substantial heterogeneity in donor body composition. Fig. 2c presents CIT, which ranged from 1 to 20 h, with a median of 8.75 h (95% CI: 4.31–13.94) and a standard deviation of 4.82 h, highlighting the variability in organ preservation duration in our study. Fig. 2d shows WIT, ranging from 10 to 120 min, with a median of 25 min ± 18.75, reflecting diverse donor organ retrieval conditions. These distributions highlight the variability in donor demographics and organ handling conditions, therefore enabling us to analyze the impact of each of these variables on the cells’ viability and functions after cryopreservation.

### Viability, cell yield, and storage characteristics of cryopreserved human hepatocytes

**Figure 3.**
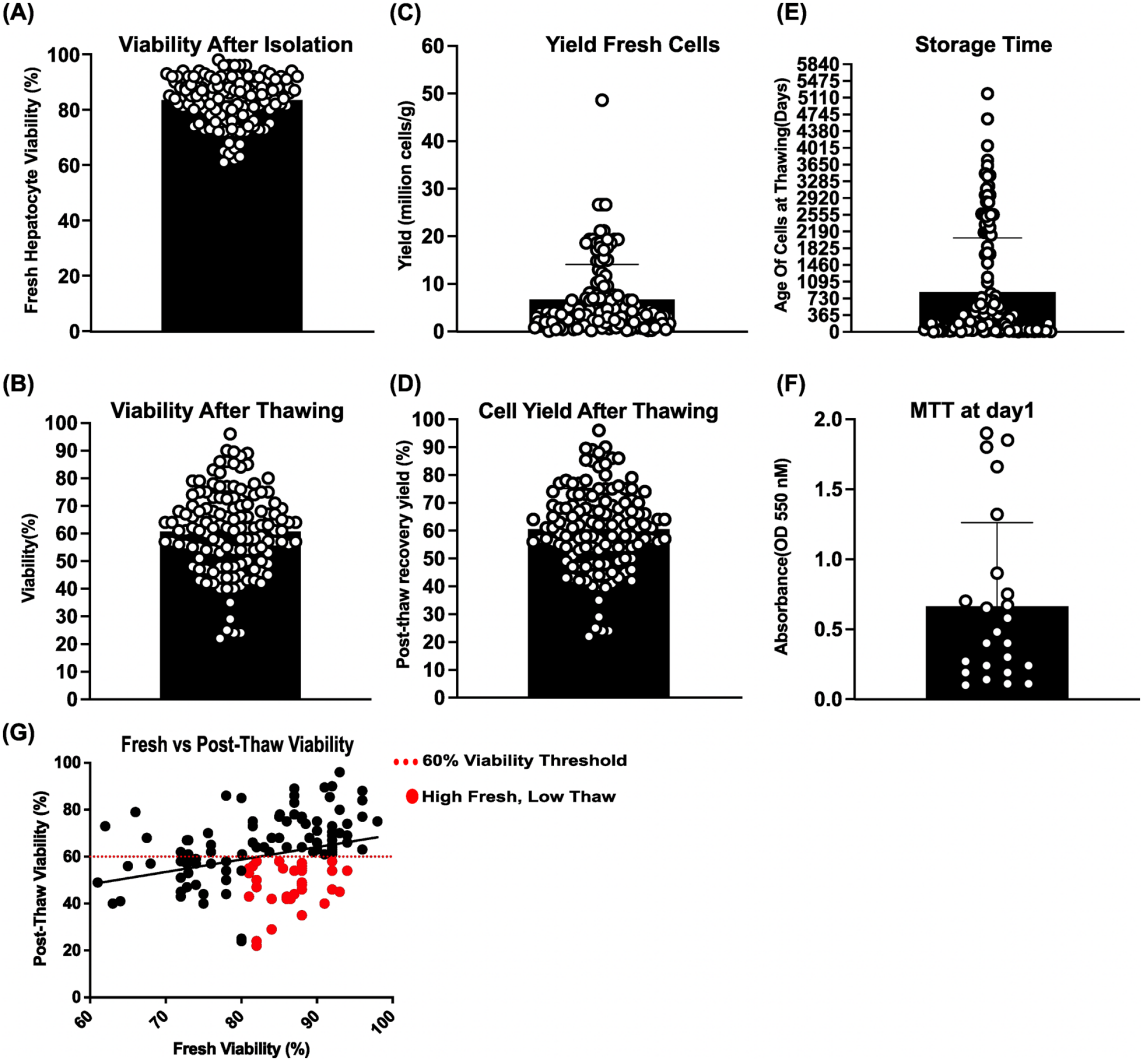
(a) Fresh hepatocyte viability (*n* = 81) at isolation was assessed by Trypan Blue exclusion (mean: 85.3% ± 8.29%; range: 61%–98%). (b) Post-thaw viability (*n* = 133), measured using the same method, decreased to a mean of 61.0% ± 14.69% (range: 22%–96%). (c) Fresh cell yield (*n* = 81), normalized to donor liver tissue weight, ranged from 0.075 to 48.60 million cells per gram (M cells/g), with a mean of 6.70 ± 7.36 M cells/g. (d) Post-thaw cell recovery yield (*n* = 107), expressed as the number of viable cells recovered per vial. (e) The storage time after cryopreservation (Age of Cells at Thawing (days). (f) Metabolic activity post-thaw (*n* = 24) was assessed using the MTT assay (OD 550 nm). (g) Correlation between fresh and post-thaw viability across 81 hepatocyte batches (*R*^2^ = 0.092, *P* = 0.0004). The red dashed line indicates the 60% clinical viability threshold. Red dots highlight batches with high fresh viability (>80%) but poor post-thaw performance (<60%), labeled as “High Fresh, Low Thaw.” Each data point represents an individual donor batch. Black bars indicate the mean ± standard deviation (SD).

Fig. 3 summarizes hepatocyte viability, cell yield, and cryopreservation-related parameters across 81 donor batches. As shown in Fig. 3a, the viability of hepatocytes at isolation was 85.3% ± 8.29% (range: 61%–98%). Following cryopreservation, post-thaw viability decreased to 61.0% ± 14.69% (range: 22%–96%) (Fig. 3b).

Fresh hepatocyte yield, normalized to liver tissue weight, ranged from 0.075 to 48.60 million cells per gram of tissue (M cells/g), with a mean yield of 6.70 ± 7.36 M cells/g (Fig. 3c). These metrics compare favorably with those reported by other laboratories using non-cryopreserved cells, indicating that our hepatocyte yields are consistent with existing data. This suggests comparable tissue quality and isolation techniques, supporting the reproducibility of our findings in other settings, particularly in laboratories not yet employing cryopreservation. In contrast, post-thaw recovery yield, defined as the percentage of viable cells recovered relative to the number originally frozen, ranged from 22.0% to 96.0%, with a mean of 60.62% ± 14.52% (Fig. 3d). Correlation analysis revealed a statistically significant but weak positive association between fresh isolation yield and post-thaw recovery yield (*R*^2^ = **0.1001**, *P* = **0.0002**), indicating that higher initial yields may modestly predict improved post-thaw outcomes (data not shown). Cryopreservation storage time spanned from 365 to 5200 days, that is, over 15 years (Fig. 3e). Our dataset includes 29 hepatocyte batches cryopreserved for more than 5 years, allowing us to robustly assess the impact of long-term storage on cell quality and function.

Out of the original 81 hepatocyte batches analyzed, a subset of 24 batches was plated and assessed for metabolic activity using the MTT assay. These batches were considered representative of the full dataset, with a post-thaw viability of 66.3% ± 13.1% (range: 42%–90%) compared with the overall mean of 61.0% ± 14.69%. The storage duration for these batches ranged from 8 to 1868 days, allowing us to assess the effects of up to 5-year cryopreservation, but not the impact of storage durations exceeding 10 years. Metabolic activity, assessed via MTT assay at day 1 post-thaw, showed a mean absorbance of 0.665 ± 0.584 OD units at 550 nm (Fig. 3f).

A paired *t*-test comparing the viability of fresh and post-thaw hepatocytes confirmed that the reduction in viability was statistically significant (*P* < 0.0001), indicating a substantial loss of viable cells due to the cryopreservation process. A scatter plot of fresh versus post-thaw hepatocyte viability (Fig. 3g) showed a significant but weak correlation (*R*^2^ = **0.092**, *P* = **0.0004**), indicating limited predictive value. Out of the 81 batches analyzed here, 34 batches (42%) would still have qualified for clinical use, based on a 60% minimum viability. Whilst 47 (58%) fell below the threshold. Surprisingly, some batches with fresh viability >80% had poor post-thaw outcomes. This prompted further analysis of donor and retrieval variables to better guide our donor selection for subsequent cell cryopreservation.

### Impact of cryopreservation storage duration on hepatocyte viability and function

To determine whether storage duration affects cell quality, we assessed the potential correlation between cell viability, metabolic activity, functional assays, and hepatocyte cryopreservation storage duration. To aid interpretation, we first note that sample sizes (*n*) vary across experiments. Exact values for each dataset are provided in the corresponding figure legends. D–F plots were generated using a limited subset of samples due to the requirement for both high-quality thawed hepatocytes and sufficient cell numbers. These samples had fresh cell viability ranging from 60% to 94% and post-thaw viability from 49% to 75%, representative of the usable donor pool.

**Figure 4.**
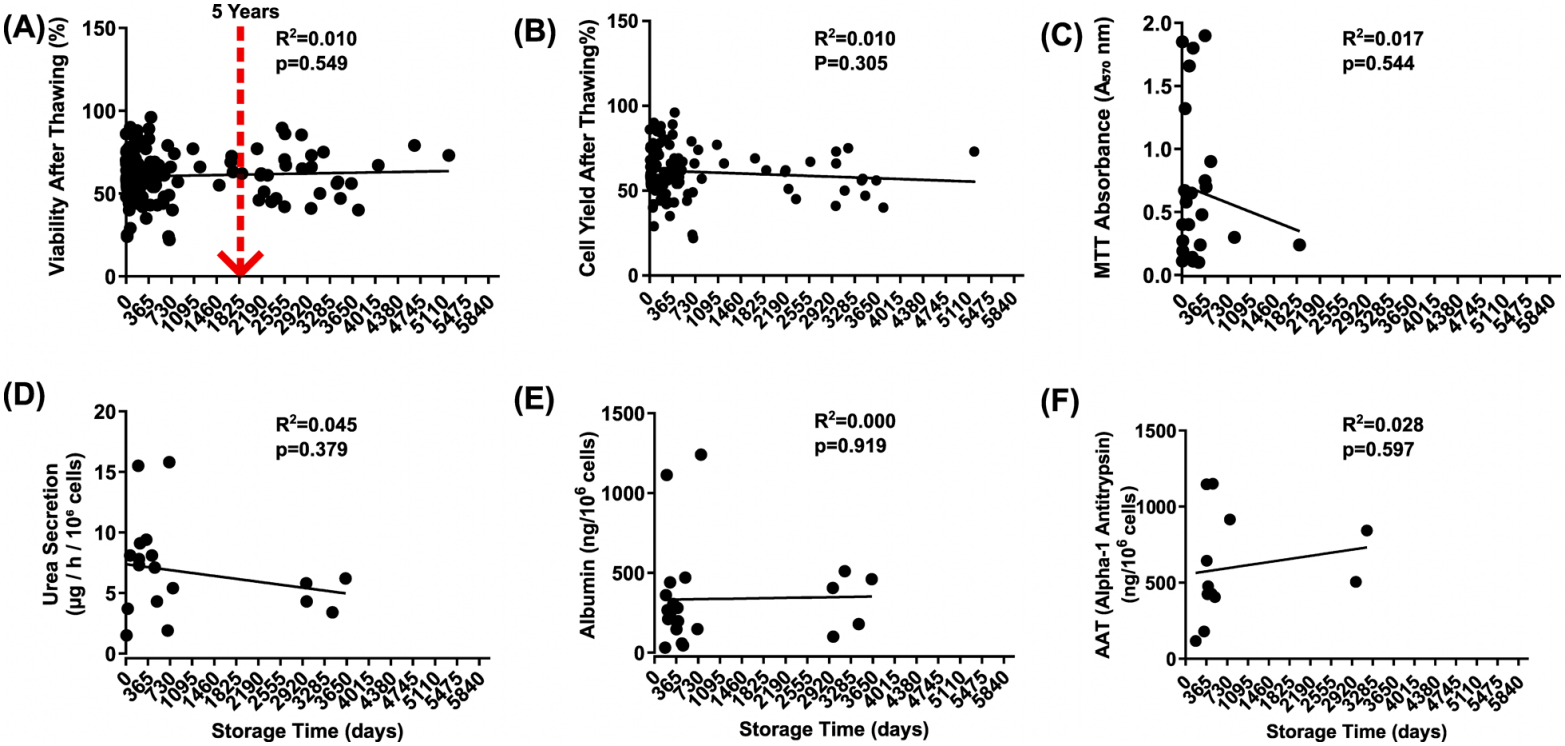
Each scatter plot (Panels a–f) illustrates the relationship between cell viability or functional parameter (y-axis) and the storage time in cryopreservation (x-axis). (a) Viability after thawing (%), the red dashed line marks a storage duration of 5 years, included here as a reference point. The horizontal red dashed line indicates the minimum acceptable post-thaw viability threshold of 60% that we need. (b) Cell yield after thawing (%), (c) MTT absorbance (A570 nm, metabolic activity), (d) urea secretion (μg/l/10^6^ cells), (e) albumin production (ng/10^6^ cells), (f) alpha-1 antitrypsin (AAT) secretion (ng/10^6^ cells). Viability (*n* = 133), yield (*n* = 107), MTT (*n* = 24), urea (*n* = 19), albumin (*n* = 22), AAT (*n* = 11).

Figure 4 presents scatter plots of each variable versus storage time, with regression lines and corresponding *r*^2^ and *P* values. Hepatocyte viability after thawing (*R*^2^ = **0.010**, *P* = **0.549**) (Fig. 4a) and cell yield (*R*^2^ = **0.010**, *P* = **0.305**) (Fig. 4b) show no significant correlation with storage time, suggesting that cryopreservation duration does not substantially impact post-thaw cell recovery. Notably, 45.6% of samples stored for 0–5 years and 52.9% of those stored for 5–15 years had post-thaw viabilities below 60%, supporting the feasibility of extending hepatocyte shelf life beyond 5 years. MTT absorbance exhibits a weak negative correlation with storage duration (*R*^2^ = **0.017**, *P* = **0.544**) (Fig. 4c), though this is not statistically significant. Urea secretion (*R*^2^ = **0.045**, *P* = **0.379**) (Fig. 4d), albumin production (*R*^2^ = **0.000**, *P* = **0.919**) (Fig. 4e), and AAT secretion (*R*^2^ = **0.028**, *P* = **0.597**) (Fig. 4f) are not significantly affected by storage duration, indicating that these hepatic functions remain relatively stable over time. These findings suggest that cryopreserved hepatocytes retain their quality up to 14 years post-thaw, with no significant impact from storage duration.

### Effect of donor age on post-thaw hepatocyte viability and function

**Figure 5.**
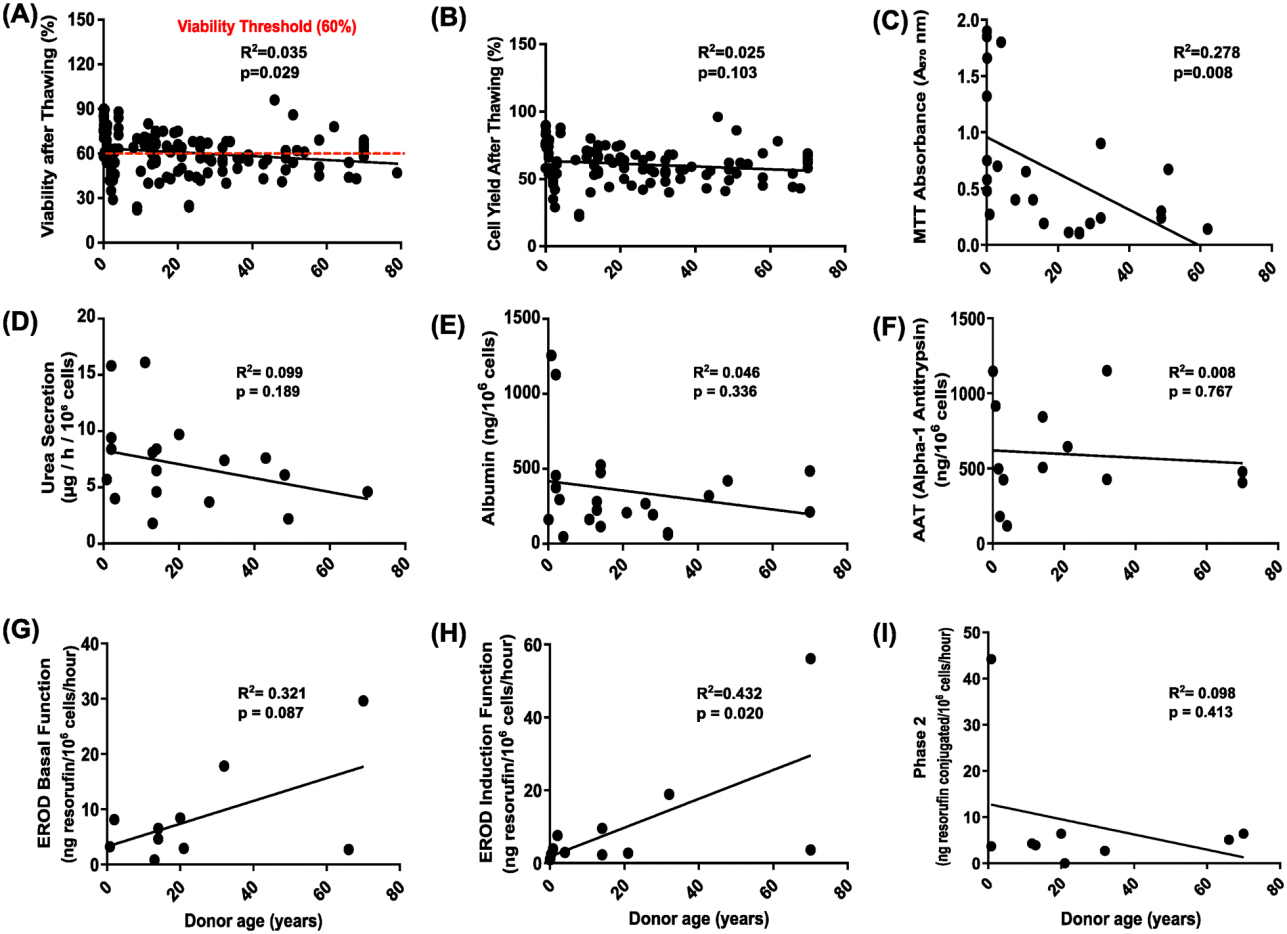
Each scatter plot (Panels a–i) illustrates the relationship between cell viability or functional parameter (y-axis) and donor age in cryopreservation (x-axis). (a) Viability after thawing (%) (*n* = 133), the red dashed line indicates our current specification for a post-thaw viability threshold of 60% in clinical cells. (b) Cell yield after thawing (%)(*n* = 107), (c) MTT absorbance (absorbance units) (*n* = 24), (d) urea secretion (μg/l/10^6^ cells) (*n* = 19), (e) albumin production (ng/10^6^ cells) (*n* = 22), (f) alpha-1 antitrypsin (AAT) secretion (ng/10^6^ cells (*n* = 13)), (g) EROD basal function (ng resorufin/10^6^ cells/h (*n* = 10)), (h) EROD induction function (ng resorufin /10^6^ cells/h) (*n* = 12), and (i) phase 2 conjugation activity (ng resorufin conjugated/10^6^ cells/h) (*n* = 9). For (d–i) plots, sample sizes per assay range from *n* = 8 to *n* = 19. *R*^2^ and *P* values indicate the strength and significance of the correlation. For each panel, the coefficient of determination (*R*^2^) and *P* values (with *P* < 0.05 considered statistically significant) are displayed to indicate the strength and significance of the correlation, while the regression line illustrates its direction.

We assessed the influence of donor age on post-thaw cell quality by examining its correlation with key cell functions/attributes (Fig. 5). Hepatocyte viability after thawing demonstrated a statistically significant, though weak, negative correlation with donor age (*R*^2^ = **0.035**, *P* = **0.029**). Although significant, the very low *R*^2^ value indicates limited explanatory power, suggesting donor age alone is insufficient as a reliable predictor of post-thaw hepatocyte viability. Based on our regression analysis, the predicted threshold for viability falling below 60% lies closer to ~45 years. However, substantial variability was observed, with multiple donors above this age still exhibiting post-thaw viabilities above the clinical acceptance threshold (Fig. 5a). Cell yield after thawing showed no significant correlation with donor age (*R*^2^ = **0.025**, *P* = **0.103**), suggesting no reduced cell recovery in older donors (Fig. 5b).

Cellular oxidative activity, assessed by the MTT assay, declined significantly with increasing donor age (*R*^2^ = **0.278**, *P* = **0.008**) (Fig. 5c), indicating an age-associated impairment in mitochondrial metabolic function. Interestingly, though, the induced EROD function showed a significant positive correlation with donor age in thawed cells (*R*^2^ = **0.432**, *P* = **0.020**) (Fig. 5h), suggesting that inducible detoxification capacity may be retained or even enhanced in hepatocytes from older donors.

With the current data we gathered, no significant correlations were observed between donor age and urea secretion, albumin production, AAT secretion, EROD basal function, or Phase 2 conjugation activity (*P* > 0.05 for all; Fig. 5d–g and i).

These results indicate that hepatocytes from older donors exhibit reduced post-thaw viability and metabolic activity, while maintaining key functions such as albumin, urea, and AAT secretion.

### Correlation between donor BMI and hepatocyte function post-thawing

**Figure 6.**
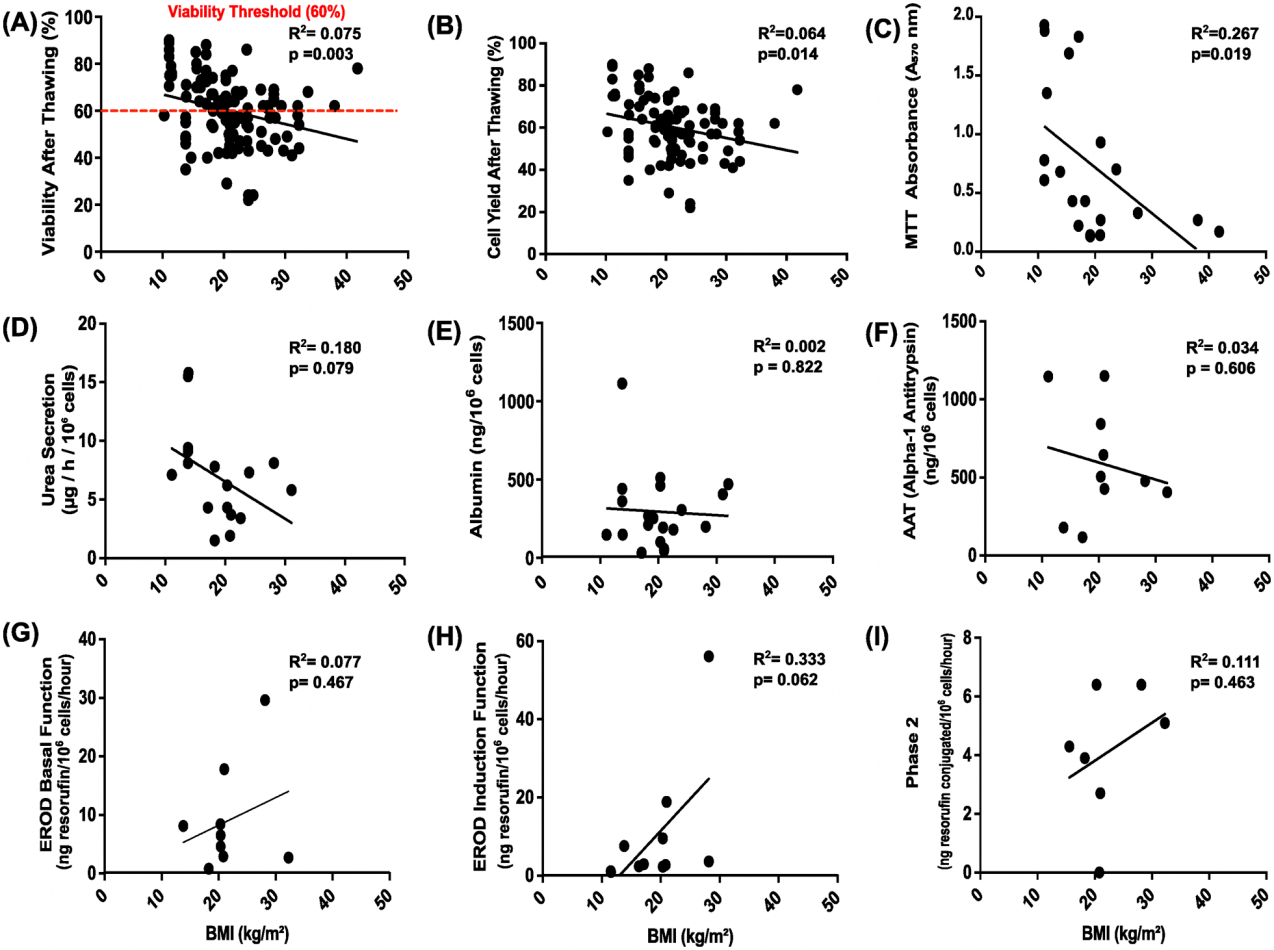
Each scatter plot (Panels a–i) represents the relationship between cell viability or functional parameter (y-axis) and BMI in cryopreservation (x-axis). (a) Viability after thawing (%) (*n* = 133), the red dashed line indicates the minimum acceptable post-thaw viability threshold of 60% that we need. (b) Cell yield after thawing (%) (*n* = 107), (c) MTT absorbance (A570 nm, metabolic activity) (*n* = 20), (d) urea secretion (μg/L/10^6^ cells) (*n* = 19), (e) albumin production (ng/10^6^ cells) (*n* = 20), (f) alpha-1 antitrypsin (AAT) secretion (ng/10^6^ cells) (*n* = 10), (g) EROD basal function (ng resorufin/10^6^ cells/h) (*n* = 9), (h) EROD induction function (ng resorufin/10^6^ cells/h (*n* = 11)), and (i) Phase 2 conjugation activity (ng resorufin conjugated/10^6^ cells/h) (*n* = 7). For (d–i) plots, sample sizes per assay range from *n* = 6 to *n* = 19. *R*^2^ and *P* values indicate the strength and significance of the correlation. For each panel, the coefficient of determination (*R*^2^) and *P* values (with *P* < 0.05 considered statistically significant) are displayed to indicate the strength and significance of the correlation, while the regression line illustrates its direction.

Our team had often observed that cells from fatty livers would tend not to withstand cryopreservation well, without formally assessing this. Due to the frequency of non-alcoholic fatty liver disease (NAFLD) in obese people, we therefore examined whether the liver donors’ BMI impacted post-thawing cell function or viability. Donor BMI exhibits varying negative associations with hepatocyte viability and functional outputs following thawing (Fig. 6). Post-thaw hepatocyte viability shows a significant negative correlation with BMI (*R*^2^ = **0.075**, *P* = **0.003**). However, this association is weak, and thus, BMI alone does not robustly predict hepatocyte viability. The 60% viability threshold is crossed at a BMI of approximately 20 kg/m^2^. However, substantial variability exists in our data, as seen with the low *R*^2^ value, and several donors with BMI above 30–40 kg/m^2^ still yielded clinically acceptable viability (Fig. 6a). Similarly, cell yield after thawing was also negatively correlated with BMI, but with a large spread in data points (*R*^2^ = **0.064**, *P* = **0.014**) (Fig. 6b), further confirming the detrimental effect of obesity on hepatocyte recovery.

Oxidative activity, as measured by MTT assay, demonstrates a strong negative correlation with BMI (*R*^2^ = **0.267**, *P* = **0.019**) (Fig. 6c), suggesting a significant decline in mitochondrial function with increasing BMI.

No significant correlations were observed between BMI and urea secretion, albumin production, AAT levels, EROD basal function, induced EROD, or Phase II conjugation (all *P* > 0.05) (Fig. 6d–i).

Taken together, these findings indicate that increased donor BMI is significantly associated with reduced post-thaw viability, cell yield, and mitochondrial metabolic activity in hepatocytes. Other functional parameters, including nitrogen metabolism, albumin production, and detoxification functions, showed no significant association with BMI.

### Impact of WIT on hepatocyte viability, metabolic activity, and post-thaw function

**Figure 7.**
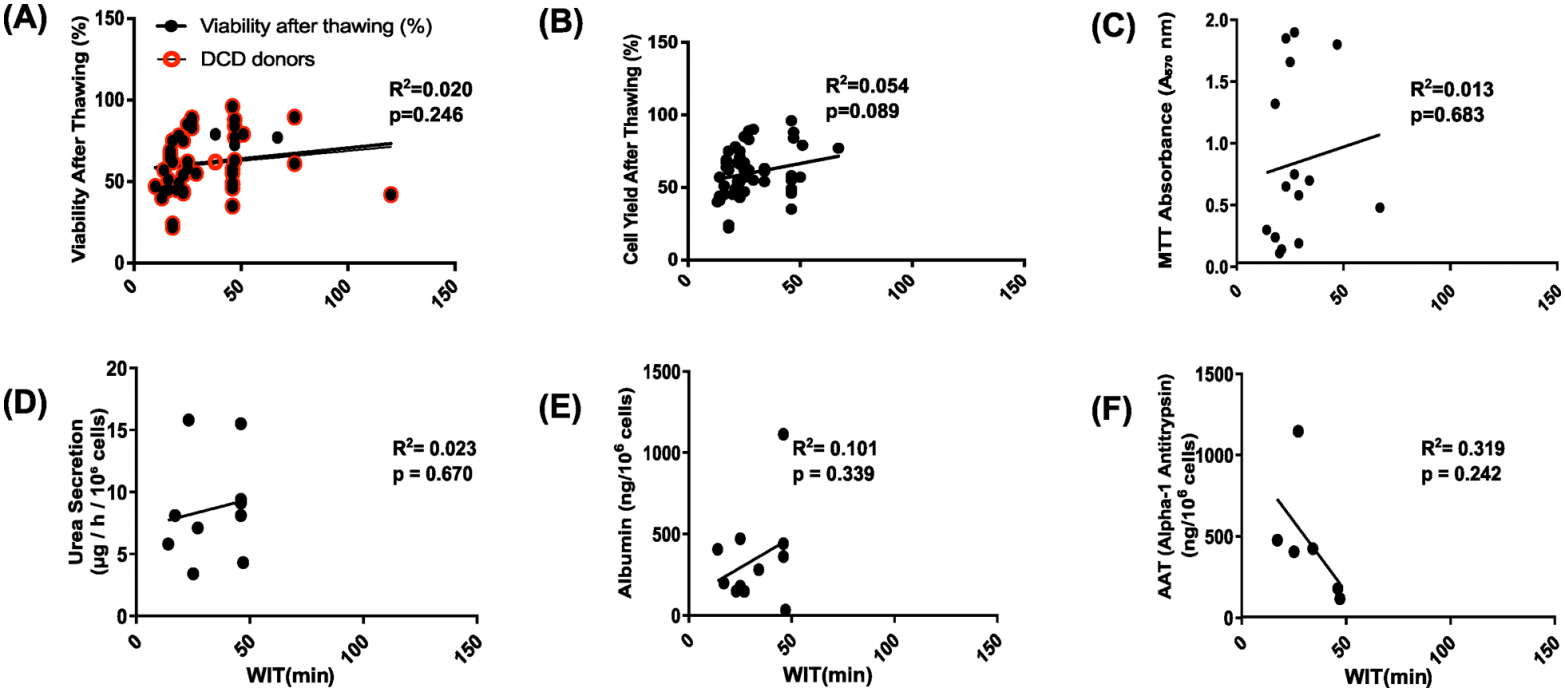
Each scatter plot (Panels a–f) illustrates the relationship between cell viability or functional parameter (y-axis) and WIT in cryopreservation (x-axis). (a) WIT correlation with post-thaw viability (*n* = 69), stratified by donor type (DCD, *n* = 45). (b) Cell yield after thawing (%) (*n* = 69), (c) MTT absorbance (A570 nm, metabolic activity) (*n* = 15), (d) urea secretion (μg/l/10^6^ cells) (*n* = 10), (e) albumin production (ng/10^6^ cells) (*n* = 11), (f) alpha-1 antitrypsin (AAT) secretion (ng/10^6^ cells) (*n* = 6).

WIT is recorded for donation after circulatory death (DCD) donors—donation after brain death (DBD) donors do not undergo warm ischemia—and is currently included as part of our tissue acceptance criteria. Donor stratification showed 61.2% DBD (*n* = 74) and 38.8% DCD (*n* = 47). Among DCD donors, the median WIT was 24.0 min. This analysis of the impact of WIT length aimed to evaluate whether our existing WIT thresholds should be revised. However, within the range of accepted WIT values, no consistent impact on post-thaw hepatocyte viability or function was observed, as shown in Fig. 7. Stratification by donor type revealed no significant association between WIT and post-thaw viability in DCD donors (*R*^2^ = **0.004**, *P* = **0.802**) nor with functional outcomes. Due to the limited number of high-WIT samples, further analysis was not pursued.

### Outcomes of CIT on post-thaw hepatocyte viability, function, and metabolic activity

**Figure 8.**
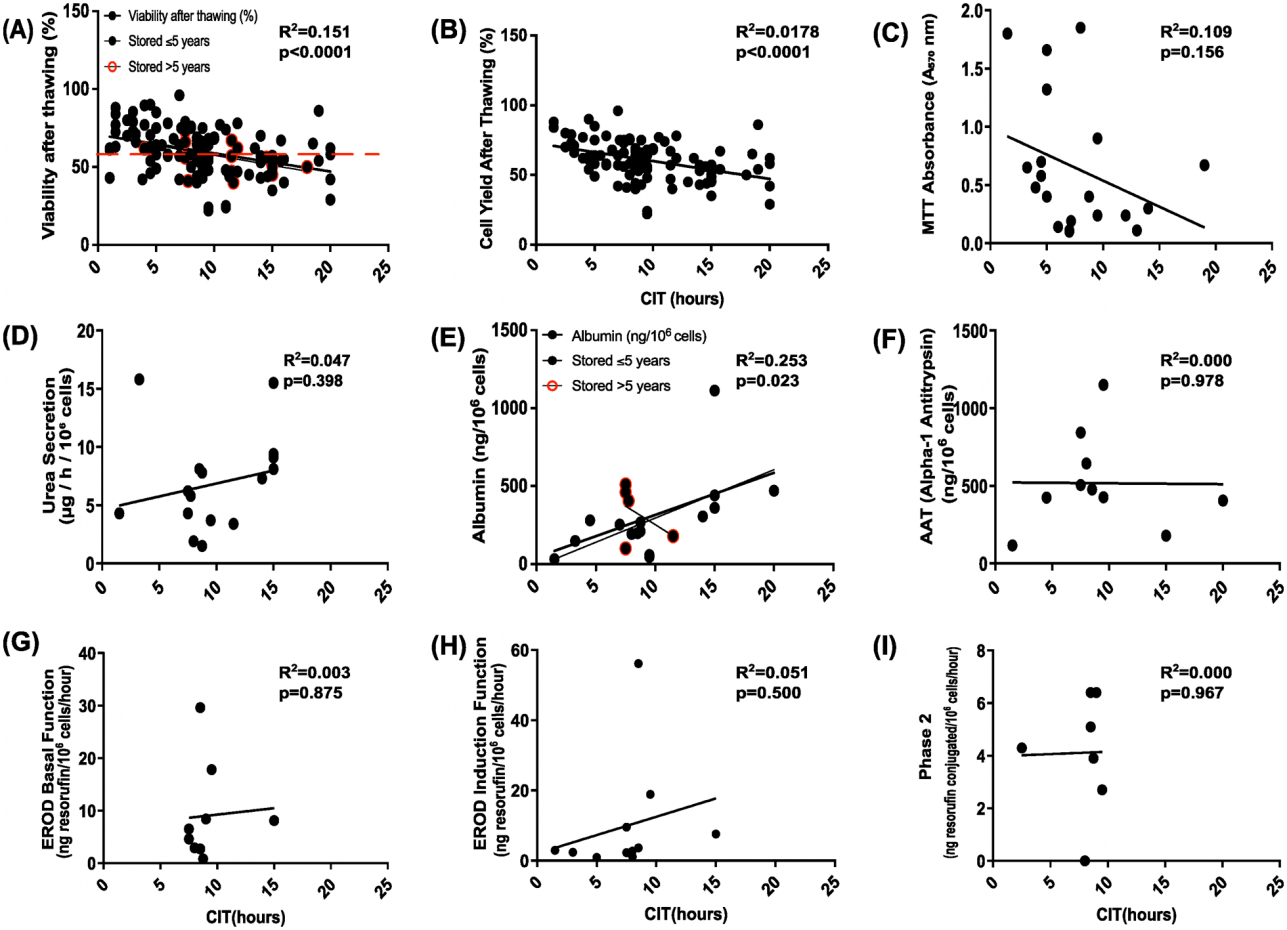
Each scatter plot (Panels a–i) illustrates the relationship between cell viability or functional parameter (y-axis) and CIT in cryopreservation (x-axis). (a) Viability after thawing (%) (*n* = 133), the red dashed line indicates the minimum acceptable post-thaw viability threshold of 60% that we need. (b) Cell yield after thawing (%) (*n* = 107), (c) MTT absorbance (A570 nm, metabolic activity) (*n* = 20), (d) urea secretion (μg/l/10^6^ cells) (*n* = 17), (e) albumin production (ng/10^6^ cells) (*n* = 20), (f) alpha-1 antitrypsin (AAT) secretion (ng/10^6^ cells) (*n* = 13), (g) EROD basal function (ng resorufin/10^6^ cells/h) (*n* = 9), (h) EROD induction function (ng resorufin/10^6^ cells/h) (*n* = 11), and (i) Phase 2 conjugation activity (ng resorufin conjugated/10^6^ cells/h) (*n* = 7). *R*^2^ and *P* values indicate the strength and significance of the correlation. For each panel, the coefficient of determination (*R*^2^) and *P* values (with *P* < 0.05 considered statistically significant) are displayed to indicate the strength and significance of the correlation, while the regression line illustrates its direction. Open red circles represent hepatocyte batches stored for more than 5 years, allowing visualization of both CIT and long-term storage effects. Regression lines are shown separately for ≤5 years (black) (*n* = 90) and >5 years (red) (*n* = 17).

The effect of CIT on post-thaw hepatocyte quality was assessed (Fig. 8). A weak but statistically significant negative correlation was observed between CIT and viability after thawing (*R*^2^ = **0.151**, *P* < **0.0001**), Fig. 8a. To understand the potential impact of storage time, we stratified these data for samples stored for more or less than 5 years. When samples had been cryopreserved for ≤5 years, there was a significant negative correlation (*R*^2^ = **0.143**, *P* < **0.0001**), suggesting that prolonged CIT reduces hepatocyte viability when they are frozen and thawed. No significant correlation was found in samples stored >5 years (*R*^2^ = 0.200, *P* = 0.0945), likely due to a smaller sample size. Notably, viability values tended to fall below the 60% threshold when CIT exceeded approximately 10 h. In our dataset, 24 batches had a CIT greater than 10 h, of which 12 (50 %) still retained post-thaw viability above 60%. Similarly, cell yield after thawing was also significantly correlated with CIT (*R*^2^ = **0.0178**, *P* < **0.0001**) in Fig. 8b.

In addition, albumin secretion showed a significant positive correlation with CIT (*R*^2^ = **0.253**, *P* = **0.023**) and in cells stored ≤5 years (*R*^2^ = **0.36**, *P* = **0.019**), Fig. 8e. Interestingly, cells stored for more than 5 years (red dots) did not appear to show reduced albumin production.

No significant correlations were observed between CIT, MTT activity, urea secretion, AAT secretion, EROD basal activity, induced EROD, or Phase II conjugation (*P* > 0.05 for all these parameters).

As summarized in Table 1, *storage time* shows no significant correlation with any of the dependent variables, indicating that within the analyzed range, cryostorage duration does not impact cell viability or function. Similarly, WIT, within our current specifications, does not correlate with any measured outcomes. In contrast, donor age exhibits a significant negative correlation with both cell recovery and post-thaw viability. CIT also demonstrates a significant negative correlation with post-thaw viability and albumin levels. In addition, donor BMI correlates negatively with cell recovery, post-thaw viability, and MTT results. Other donor and tissue parameters did not show any significant association with the analyzed outcomes. However, a weak but statistically significant correlation was observed between fresh cell viability and post-thaw viability (*R*^2^ = **0.092**, *P* = **0.0004**) (data not shown), suggesting that higher initial viability may modestly predict post-thaw performance.

**Table 1.** Correlation coefficients *R*^2^ and *P* values for all the linear regression analyses performed.

		Independent variables				
		Storage time	Age of donor	WIT	CIT	BMI
Dependent variables	% Viability (Trypan blue) *n* = 133	*R*^2^ = 0.010 *P* = 0.549	*R*^2^ = 0.035 *P* = 0.029*	*R*^2^ = 0.020 *P* = 0.246	*R*^2^ = 0.151 *P* < 0.0001**	*R*^2^ = 0.075 *P* = 0.003*
	Cell yield after thawing (%) *n* = 107	*R*^2^ = 0.010 *P* = 0.305	*R*^2^ = 0.025 *P* = 0.103	*R*^2^ = 0.054 *P* = 0.89	*R*^2^ = 0.001 P<0.0001*	*R*^2^ = 0.069 *P* = 0.011*
	MTT at day 1 after thawing/plating *n* = 24	*R*^2^ = 0.017 *P* = 0.544	*R*^2^ = 0.278 *P* = 0.008**	*R*^2^ = 0.013 *P* = 0.683	*R*^2^ = 0.109 *P* = 0.156	*R*^2^ = 0.267 *P* = 0.019**
	Albumin *n* = 22	*R*^2^ = 0.000 *P* = 0.919	*R*^2^ = 0.046 *P* = 0.336	*R*^2^ = 0.101 *P* = 0.339	*R*^2^ = 0.253 *P* = 0.023**	*R*^2^ = 0.002 *P* = 0.822
	AAT *n* = 13	*R*^2^ = 0.028 *P* = 0.597	*R*^2^ = 0.008 *P* = 0.767	*R*^2^ = 0.319 *P* = 0.242	*R*^2^ = 0.000 *P* = 0.978	*R*^2^ = 0.034 *P* = 0.606
	Urea secretion *n* = 19	*R*^2^ = 0.045 *P* = 0.379	*R*^2^ = 0.099 *P* = 0.189	*R*^2^ = 0.023 *P* = 0.670	*R*^2^ = 0.047 *P* = 0.398	*R*^2^ = 0.180 *P* = 0.079
	Basal EROD *n* = 10	*R*^2^ = 0.023 *P* = 0.673	*R*^2^ = 0.321 *P* = 0.087	*R*^2^ = 0.255 *P* = 0.662	*R*^2^ = 0.003 *P* = 0.875	*R*^2^ = 0.077 *P* = 0.467
	Induced EROD *n* = 12	*R*^2^ = 0.002 *P* = 0.879	*R*^2^ = 0.432 *P* = 0.020	*R*^2^ = 0.131 *P* = 0.549	*R*^2^ = 0.051 *P* = 0.500	*R*^2^ = 0.333 *P* = 0.062
	Phase 2 *n* = 9	*R*^2^ = 0.108 *P* = 0.385	*R*^2^ = 0.098 *P* = 0.413		*R*^2^ = 0.000 *P* = 0.967	*R*^2^ = 0.111 *P* = 0.463

*Note*. The values highlighted refer to correlations considered statistically significant. **indicates strong and statistically significant correlations (|*R*| > 0.7, *P* < 0.05). *shading indicates weak but statistically significant correlations (|*R*| ≤ 0.7, *P* < 0.05). Non-significant correlations (*P* ≥ 0.05) are not shaded.

Table 1, storage time, shows no significant correlation with any of the dependent variables, indicating that within the analyzed range, cryostorage duration does not impact cell viability or function. Similarly, WIT, within our current specifications, does not correlate with any measured outcomes. In contrast, donor age exhibits a significant negative correlation with both cell recovery and post-thaw viability. CIT also demonstrates a significant negative correlation with post-thaw viability and albumin levels. In addition, donor BMI correlates negatively with cell recovery, post-thaw viability, and MTT results. Other donor and tissue parameters did not show any significant association with the analyzed outcomes. However, a weak but statistically significant correlation was observed between fresh cell viability and post-thaw viability (*R*^2^ = **0.092**, *P* = **0.0004**) (data not shown), suggesting that higher initial viability may modestly predict post-thaw performance.

## Discussion

Our team has been isolating PHHs for clinical applications since the early 2000s. We have successfully used both fresh and cryopreserved cells for the treatment of patients with inborn errors of liver metabolism or acute liver failure, originally setting a maximum shelf life for cryopreserved hepatocytes of 5 years^8,25–32^. The purpose of this study was to update our data on cryopreserved hepatocyte shelf life and analyze the impact of the liver donors’ characteristics, and the retrieval technical details, on the subsequent cell functions after thawing. Importantly, the donor characteristics in this study mirror those used in clinical hepatocyte transplantation (HTx) with freshly isolated cells, enhancing the relevance of our findings. All the batches analyzed here had an original fresh cell viability >60%, making them suitable for clinical use. We found that long-term cryopreservation up to 14 years does not impair cell viability or function. CIT, donor age, and BMI were key factors associated with post-thaw outcomes, while WIT and prolonged storage duration did not appear to adversely affect hepatocyte performance.

### Cryopreservation for up to 14 years does not affect PHHs

While hepatocyte quality after <5 years of cryopreservation is well documented, long-term effects beyond 5 years have not been fully addressed in clinically relevant settings. By including cells stored for over 5 years, we assessed how donor or tissue collection factors (age, BMI, WIT, CIT) and storage duration influenced post-thaw viability and metabolic function. Notably, cryopreservation for up to 14 years did not significantly impact these parameters, supporting the long-term storage of GMP-compliant biobanked hepatocytes in clinical settings. This aligns with Sudo’s team^33^, who showed CYP3A4/5 induction remained intact in cells stored for over a decade. Donato and colleagues^1^ similarly reported maintained *in vivo* function in short-term cryopreserved hepatocytes. To our knowledge, this is the first time anyone has reported such an extended storage time for PHHs produced for clinical applications. Interestingly, cells stored >5 years (red dots) did not show reduced albumin production, indicating that storage time alone does not impair this functional parameter.

### CIT strongly correlates with post-thaw viability, but WIT has a limited impact

CIT showed a significant inverse correlation with post-thaw viability (*R*^2^ = 0.151, *P* < 0.0001), albumin secretion, and post-thawed cell recovery, consistent with prior studies^17^. In our cohort, 24 batches had CIT >10 h, and 12 (50%) still maintained >60% viability after cryopreservation and thawing. This indicates that while CIT is a critical factor, revising current acceptance thresholds should be done cautiously to avoid excluding usable batches. When possible, however, the cell isolation process should be done as early as possible. The positive correlation between CIT and albumin synthesis may suggest selective survival of peri-portal albumin-producing cells. Stable MTT, urea, and EROD values across CIT ranges further support hepatocyte resilience.

In contrast, WIT for DCD donors—within our defined criteria of <20 min for adult donors, extended to 70 min for neonatal livers^32^—showed no significant correlation with post-thaw cell viability, yield, or metabolic activity, suggesting minimal impact on hepatocyte quality. This aligns with other studies showing preserved function in cryopreserved neonatal hepatocytes despite extended WIT^32,34,35^.

### Donor BMI negatively affects cryopreserved cell oxidative function

Liver donor BMI correlation with cell viability after cryopreservation was included in our study because it may influence hepatocyte fat content and metabolic stress^36^, which can affect hepatocyte resilience to cryopreservation and post-thaw recovery.

Donor BMI showed a modest negative correlation with viability and MTT, although the *R*^2^ was low. This differs from studies on fresh hepatocytes reporting minimal BMI impact^16,37^. Despite decreased metabolic activity in cells from higher BMI donors, protein secretion remained stable, supporting clinical utility. Among the 43 donor livers with a BMI >20, 21 (48.84%) still achieved post-thaw viability >60%. This indicates that nearly half of the high-BMI donors provided cells considered clinically viable. Given this distribution and the wide data spread, BMI alone does not reliably predict poor outcomes. Given this distribution and the weak correlations observed, BMI alone is insufficient as a reliable predictor or exclusion criterion.

### Donor age affects post-thaw metabolic performance

For freshly isolated cells, donor age has previously been limited to 70 years as the cell’s original viability decreases after that point^38^. However, we had not yet evaluated its incidence on cell viability post-cryopreservation. In thawed cells, donor age was associated with reduced MTT and yield, confirming earlier reports on cryopreserved hepatocytes^19,32,38^. However, the very low *R*^2^ value for viability (*R*^2^ = 0.035) indicates limited explanatory power, suggesting that donor age alone is insufficient as a reliable predictor of hepatocyte quality. Moreover, albumin and urea secretion were maintained across age groups. The increase in induced EROD activity with age may reflect a compensatory mechanism. However, since hepatocytes do not undergo adaptation during cryopreservation, this increase is more likely due to a selection bias, whereby cells with higher inducible CYP activity are preferentially preserved or recover better post-thaw. Among the 18 donor batches with age >40 years, 10 (55.56%) retained post-thaw viability above 60%, our threshold to use the cells in cell therapy treatments. While cells from older donors may display lower oxidative metabolic output, they remain a suitable selection for clinical transplantation. For whole organ transplantation, Lué et al.^39^ reported that although graft failure and biliary complications were more frequent, overall patient survival was not significantly compromised when using livers from older donors, in the absence of additional risk factors.

Our analysis suggests that oxidative function (MTT) declines with age, in contrast to our data showing preserved CYP450 activity in older donors. This discrepancy may stem from a very low sample size (*n* = 12) or cryopreservation-specific effects and warrants further investigation.

### Fresh versus post-thaw viability

Our criteria for clinical cell batches demand that fresh cell viability be over 60%. However, we hypothesized that cell suspensions of higher original viability may withstand cryopreservation better and result in better post-thaw viability. We therefore explored whether post-thaw cell viability correlated with fresh viability at the time of isolation. A weak but significant correlation was found (*R*^2^ = 0.092, *P* = 0.0004), indicating fresh viability may slightly predict post-thaw outcome. This could inform future cryopreservation strategies, such as prioritizing high-viability batches.

Surprisingly, 47 out of 81 batches (58%) fell below the 60% post-thaw viability threshold despite meeting clinical criteria when fresh. Notably, some of these underperforming batches originally had viability above 80%, indicating that high fresh viability does not guarantee successful recovery from cryopreservation (Fig. 3). This variability warrants further investigation into additional donor-related variables to better inform donor selection strategies for cryopreserved hepatocyte use.

### Study limitations and future directions

Our data is a retrospective analysis of data gathered by various team members over the past 14 years, while they were working on various research projects, which explains the variable n number on each set of data. The most important data for our clinical work is (1) the cell viability because this is the main (and immediate) assay that can discriminate good quality cell batches, as it is strongly correlated to cell function for fresh cells^18^; (2) synthetic and detoxification function (ALB, AAT secretion, and CYP functions and inducibility). It would have been interesting to obtain more/different information on these cells such as the impact of the cryopreservation on the subsequent identity/function stability of the cells over the following few days/weeks, when cultured in normal media or serum from patients with acute liver failure (ALF), the cell mitochondrial oxidative phosphorylation, any apoptosis induction, the impact of micro- or macro-vesicular steatosis, bile acid metabolism or transport activity.

MTT served as an indirect indicator of mitochondrial activity, but this is a very rough measurement. A separate study from our group did find immediate deleterious effects of cryopreservation on hepatocyte oxidative phosphorylation (not published). Other studies had similar results^15,40^. We do not believe that mtDNA analysis would have shown immediate post-thaw differences, but it is possible that the capacity of cells/their mitochondria to recover from cryopreservation may be altered by the length of storage and the steatotic status of these cells. The observed reduction in MTT activity with increasing donor BMI and age implies compromised post-thaw metabolic function, highlighting the need for future studies employing direct mitochondrial analyses.

A limitation of this study is the absence of an analysis of the cell stability after thawing. All our data were generated at day 1 post-thawing. Hepatocyte-specific marker analysis (e.g., HNF4α, ASGR1, ALB, BSEP, MRP2 expression) as well as functional assays (albumin secretion, ureagenesis, CYP450, or bile canalicular assays) could serve as indicators of stability and further assess hepatocyte maturity and metabolic competence. In addition, certain potentially confounding variables, including donor cause of death, the degree of macro-vesicular steatosis, and ischemia-reperfusion injury, were not consistently documented in our retrospective dataset. Future prospective studies incorporating these parameters may provide further insights into factors influencing post-thaw hepatocyte outcomes. Histological assessment of hepatic fat content (macro-vesicular and micro-vesicular steatosis) was not consistently available in this retrospective dataset. While BMI was included as a surrogate marker, future studies should incorporate direct quantification of steatosis, given its known impact on thermal conductivity, ice nucleation, and post-thaw hepatocyte viability and function. Finally, future improvements in hepatocyte preservation may benefit from alternative cryopreservation strategies such as vitrification, which has shown enhanced post-thaw viability and structural integrity in microencapsulated hepatocytes^24,40^.

## Conclusions

The aim of our study was twofold: (1) to assess the shelf life of cryopreserved hepatocytes and (2) to potentially refine our donor liver criteria for cells aimed to be cryopreserved rather than being used fresh. Our study shows that long-term cryopreservation (up to 14 years) does not compromise hepatocyte viability, supporting its clinical utility. Interestingly, the original fresh cell viability appeared to be a poor predictor of subsequent thawed cell viability.

Thawed cell quality appeared most influenced by CIT, donor BMI, and age, while WIT and storage duration showed minimal impact. These findings support the use of evidence-based donor selection criteria to optimize the yield and quality of hepatocytes selected for cryopreservation and transplantation.
